# Towards a science of rabies elimination

**DOI:** 10.1186/2049-9957-2-22

**Published:** 2013-10-02

**Authors:** Jakob Zinsstag

**Affiliations:** 1 Swiss Tropical and Public Health Institute, Associated Institute to the University of Basel, PO Box CH-4002, Basel, Switzerland

**Keywords:** Rabies, Dog, China, Control, Elimination

## Abstract

Wenwu Yin and co-workers conducted a systematic review on challenges and needs to eliminate rabies in China (Yin et al., 2013 in this journal). Their analysis shows that there is considerable overrepresentation of laboratory and basic epidemiology research. On the other hand, information on effective control activities and policies are nearly absent. Currently we know enough to control and eliminate dog rabies effectively. Continuing basic research while not engaging in the control of rabies appears almost cynical. Why is it not attractive to do research on effective control and elimination? Let us move now from the biological understanding to the science of rabies elimination.

## Multilingual abstracts

Please see Additional file 1 for translations of the abstract into the six official working languages of the United Nations.

## Background

Scientific inquiry generates new knowledge and establishes an evidence-base on which modern society relays for informed decisions and, ultimately, action [1]. Currently there is an exponential growth of accumulated knowledge, making it harder and harder to reach the edge of understanding, also coined “the burden of knowledge” (Benjamin Jones, Northwestern University, Chicago IL, USA, unpublished). One would argue that with so much accumulated knowledge and an ever deeper understanding, its translation into action and improvement of human health and wellbeing would be accelerated. However, there is a huge, and perhaps widening gap between knowledge and action, or rather the lack thereof. This issue is convincingly documented by Wenwu Yin and co-workers, who conducted a systematic review and analysed the scientific literature on rabies in the People’s Republic of China to examine challenges and needs to eliminate rabies in the country ([2] in this journal).

## Main text

**Figure 1:**
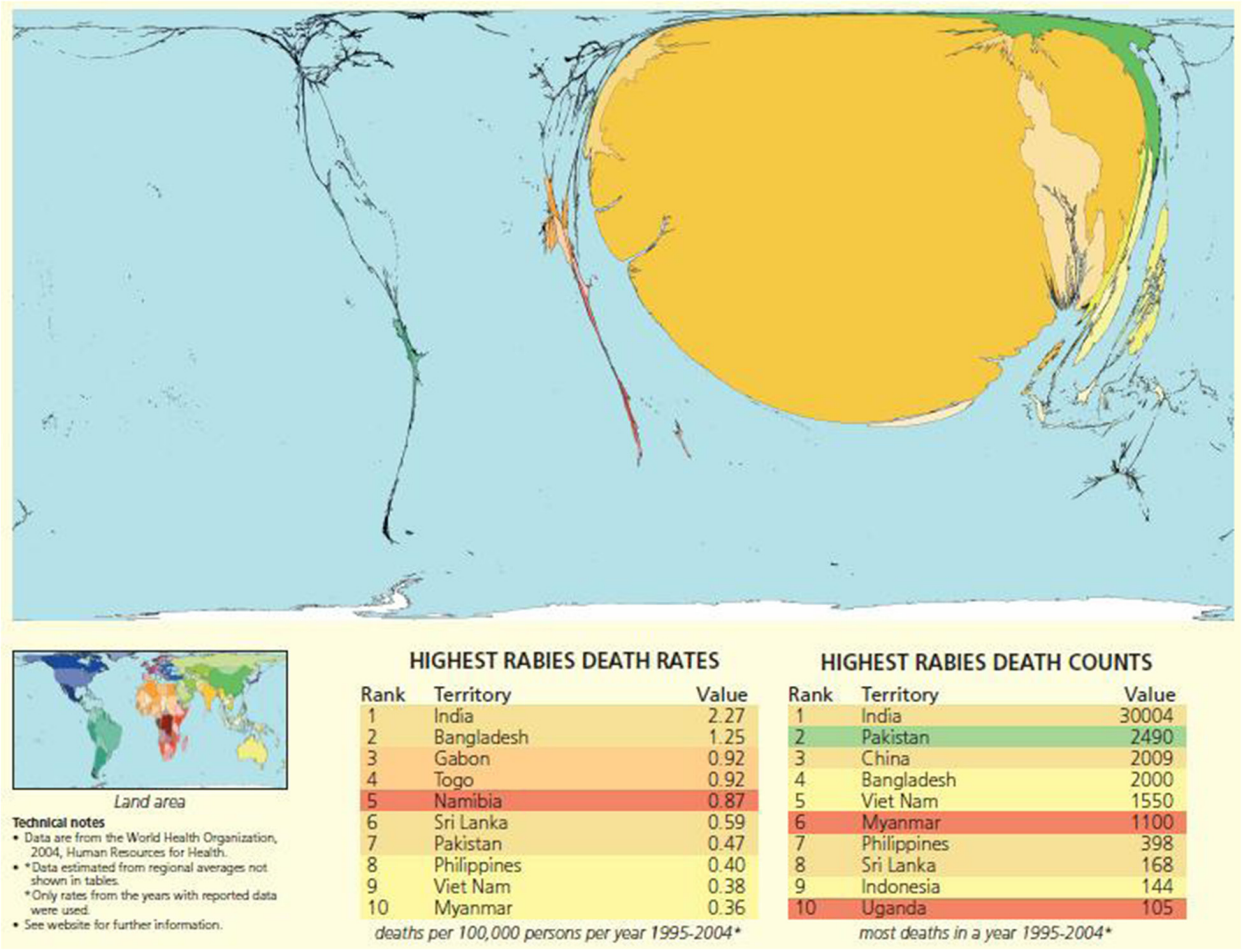
**World map of rabies death Territory size shows the proportion of human deaths from rabies worldwide that occurred there between 1995 and 2004.** © Copyright Sasi Group (University of Sheffield) and Mark Newman (University of Michigan).

In the case of dog rabies, basically all is known about the biology of rabies to effectively implement elimination [3]. To wit, highly efficacious vaccines are available for dogs. Dog rabies has been eliminated in large parts of the industrialized countries in Europe and North America. In the last decades, a concerted effort of South and Central American countries has strongly reduced dog rabies close to elimination [4]. Dog rabies persists and has even re-emerged in Asia and Africa where still more than 60′000 people die annually from this preventable disease. The largest part of the burden is borne by India and South East Asian countries followed by China (Figure 1) [5]. Using dog-human transmission models, it has been shown that dog mass vaccination leads to rabies elimination and is more cost-effective than exclusive implementation of human post exposure prophylaxis [6]. The basic reproductive ratio of dog rabies is less than 2, making it an ideal candidate for worldwide elimination [7], a goal that is pursued by the partners for rabies prevention http://www.rabiesalliance.org.

However, why is there no or insufficiently effective action to control and eliminate dog rabies? Yin’s paper, based on a systematic analysis of published literature and official documents shows that there is considerable overrepresentation of laboratory investigations or pathogen-associated and basic epidemiology research. On the other hand, information on effective control activities and policies are nearly absent. There is a lack of awareness in the general population about rabies and how to handle exposure to rabies suspected dog bites. Albeit some progress, there is still a lack of standardization of post exposure treatment in China, making it the worlds largest market for human rabies vaccination with no effect on interrupting transmission. Such practices benefit large drug companies but not the poor dwellers, who have to spend the equivalent of several months income on the cost of post exposure prophylaxis. Surveillance in dogs and in humans reports a fraction of the suspected numbers of cases and there is still a lack of communication between the public health and veterinary sector on the subject. Why is it not attractive to do research on effective control and elimination? Rolling out large-scale campaigns against dog rabies is as complex as systems biology at the subcellular level. Reaching sufficient coverage to interrupt virus transmission requires an in-depth understanding of delivery, availability, accessibility, affordability, adequacy, acceptability and many other effectiveness factors [8-10]. The article by Yin and co-workers shows an appalling situation of research still focussing on the biology of rabies rather than on the community-effectiveness of interventions. This observation is not unique for rabies, but in the case of rabies it appears almost cynical. There is a huge potential for research into the determinants of how dog rabies can be effectively eliminated. For a broad range of zoonoses and neglected tropical diseases, scientists seem to prefer sophisticated molecular analyses over investigating in effective interventions in communities. Why are research funding agencies much less interested into research aiming at effectiveness determinants rather than the biology of pathogens?

## Conclusion

Research should contribute to fill the gap between knowledge and effective action by addressing the social, political, economic and psychological complexity of effective rabies control interventions. This will require that scientists closely collaborate with authorities and communities as partners in a transdisciplinary way [11,12]. Interestingly, the study of transmission dynamics during elimination yields new insights into fundamental parameters of pathogen virulence. National governments should recognize that freedom of dog rabies is a public good, for which public funds should be invested without delay [10]. Let us move now from the biological understanding to the science of rabies elimination.

## Competing interests

The author declares that they have no competing interests.

## Supplementary Material

Additional file 1 Multilingual abstracts in the six official working languages of the United Nations. Click here for file
