# Supplementary material for: Towards a science of rabies elimination
Source: Infect Dis Poverty. 2013 Oct 2;2:22. doi: 10.1186/2049-9957-2-22 (PMC3854129; doi:10.1186/2049-9957-2-22)
Supplement: Additional file 1 — Multilingual abstracts in the six official working languages of the United Nations. [file 2049-9957-2-22-S1.pdf]

Translation of the abstract into the six official working languages of the United Nations

نحو علم يخلصنا من مرض سعار الكلب

جاكوب زنستاج

مخلص

أدار ونوو ين ومعاونيه مراجعة تنظيمية حول التحديات التي تواجه دولة الصين لمواجهة مرض سعار الكلب واحتياجاتها (ين ومعاونيه، بنشرة 2013). أظهر هذا التحليل وجود أفراط في عرض الأبحاث المعلمية وأبحاث علم الأوبئة. على الصعيد الأخر، هناك نقص ملحوظ بالمعلومات المتعلقة بنشاطات التحكم بالمرض والسياسات المنظمة لها. إن استمرار الأبحاث الأساسية دون الانخراط بالتحكم الفعلي لمرض سعار الكلب لهو أمر مثير للسخرية. لم لا يجذب البحث عن التحكم الفعال والتخلص منه الكثير من الباحثين؟ فعلينا أن ننتقل من الفهم البيولوجي إلى علم التخلص من مرض سعار الكلب.

Translated from English version into Arabic by Laila Mostafa, through

TRANSLATORS
WITHOUT BORDERS

科学迈向消除狂犬病

Jakob Zinsstag

摘要

Yin 以及团队对中国消除狂犬病的挑战与需求进行了系统综述（Yin 等，2013 年）。他们的分析结果提示已有大量的实验室和基础性流行病学研究。但另一方面，仍缺乏关于有效控制工作与政策方面的信息。目前，我们已足够掌握了有效控制与消除狂犬病知识。似乎不能想像仅开展基础性研究而不做控制狂犬病的研究。为什么不能吸引控制和消除狂犬病的研究工作呢？现在让我们从生物学研究迈向狂犬病消除科学。

Translated from English version into Chinese by Zhou Xiao-nong, through

CHINESE CENTER FOR DISEASE CONTROL AND PREVENTION
NATIONAL INSTITUTE FOR PARASITIC DISEASES

Pour une science tendant vers l'élimination de la rage

Jakob Zinsstag

Abstract

Wenwu et ses collègues ont passé en revue de manière systématique la littérature concernant les défis et les besoins liés à l'élimination de la rage en Chine (Yin et al., 2013 dans le présent journal). Selon leur analyse, il existe une surreprésentation des données de recherche épidémiologique, qu'elle soit fondamentale ou effectuée en laboratoire. Par contre, on dispose de peu de données sur les politiques et mesures adoptées pour contrôler cette maladie de manière efficace. Grâce aux connaissances actuelles, on est suffisamment équipé pour contrôler et enrayer adéquatement la rage chez le chien. Continuer la recherche fondamentale et négliger les activités de contrôle, cela relève presque du cynisme. N'est-ce pas intéressant de s'engager dans la voie de la recherche axée sur le contrôle et l'élimination définitive de la rage? Il est temps de passer d'une compréhension de la biologie à une science tendant vers l'élimination de la rage.

Translated from English version into French by Leibnitzl, through

TRANSLATORS
WITHOUT BORDERS

На пути к излечению гидрофобии

Джейкоб Зинсстаг

Краткий обзор

Венву Ин с коллегами проводили систематический анализ проблем и потребностей по излечению гидрофобии в Китае (Ин и др., 2013 год). Проведенный анализ показал, что количество осуществленных лабораторных и базовых эпидемиологических исследований достаточно велико. С другой стороны, данные об эффективных мерах и политике контроля распространения заболевания практически отсутствуют. В настоящий момент мы обладаем достаточной информацией, чтобы эффективно контролировать и искоренять случаи собачьего бешенства. Продолжать базовые исследования, не контролируя распространение заболевания, — это, по меньшей мере, цинично. Почему бы не провести исследования мер эффективного контроля и искоренения гидрофобии? Давайте, наконец, перейдем от биологического понимания к научной основе излечения гидрофобии.

Translated from English version into Russian by Irina Zayonchkovskaya, through

TRANSLATORS
WITHOUT BORDERS

Aproximación a una ciencia de erradicación de la rabia

Jakob Zinsstag

Resumen

En un análisis sistemático de los problemas y necesidades a la hora de erradicar la rabia en China (Yin *et al*., 2013 en la presente revista), llevado a cabo por Wenwu Yin y sus colaboradores, se pone de manifiesto que existe una representación excesiva de las investigaciones de laboratorio o de epidemiología básica, mientras que la información sobre directrices y actividades de control eficaces es prácticamente inexistente. En la actualidad, tenemos conocimientos suficientes para controlar y erradicar eficazmente la rabia canina. Por ello, proseguir con las investigaciones básicas sin acometer el control de dicha enfermedad resulta un tanto cínico. ¿Por qué no existe un interés por investigar la erradicación o control efectivos de esta? Avancemos ahora más allá del conocimiento biológico para pasar a una ciencia de erradicación de la rabia.

Translated from English version into Spanish by Elisa Martinez-Aznar, through

TRANSLATORS
WITHOUT BORDERS
